# Resolving a Multi-Generational Neuromuscular Mystery in a Family Presenting with a Variable Scapuloperoneal Syndrome in a c.464G>A, p.Arg155His *VCP* Mutation

**DOI:** 10.1155/2019/2403024

**Published:** 2019-10-09

**Authors:** Nivedita U. Jerath

**Affiliations:** AdventHealth Orlando, Neuromuscular Division, 1573 West Fairbanks, Winter Park, FL 32789, USA

## Abstract

Valosin containing protein (VCP) mutations have been reported to present with a high degree of variability and can be present in patients even if they may have an initial normal work up. A 55-year-old woman was labeled as “normal” and “pain medication seeking” after an unrevealing work up of clinical, laboratory, electrodiagnostic, radiographic, pathologic, and genetic testing. She continued to present with chronic neck pain, and had variable features of scapuloperoneal atrophy, which was also seen in her family. The patient and her family were found to have a known pathogenic c.464G>A, p.Arg155His (R^155^H) mutation in the *VCP* gene. Despite traditional thinking of attempting to localize neurological syndromes, *VCP* mutations are difficult to localize as they can present with significant clinical heterogeneity including a scapuloperoneal syndrome with variable neuropathic and myopathic features.

## 1. Introduction

The valsoin-containing protein (*VCP)* can manifest variable clinical phenotypes due to its regulation of several distinct cellular processes [1]. It has even been called a “jack of all trades” in neuro and myodegeneration [2]. Although previously *VCP* mutations were known to cause hereditary inclusion body myopathy (IBM), Paget's disease of the bone (PDB), and frontotemporal dementia (FTD), collectively known as IBMFTD [1], there are now reported cases of familial amyotrophic lateral sclerosis (ALS), hereditary spastic paraplegia (HSP), parkinsonism, and Charcot-Marie-Tooth Type 2 disease [3], thus confirming that *VCP* mutations are multisystem proteinopathies (MSP) [4]. Although there is such significant variability in *VCP* mutations and clinical presentations [5, 6], the disease is inherited in an autosomal dominant fashion.

## 2. Case Report

The proband is a 55-year-old Caucasian woman of Cornelius decent who was the product of a normal pregnancy and delivery. She reached her milestones on time. As a child, she was a fast runner for short distances, but could not run long distances or jump hurdles. She could ride a bike, but she could not roller skate. She could not walk on a log over a stream; she was noted to be very clumsy and had trouble with balance. She was unable to wear high heels and would have multiple falls due to weak ankles.

She would work every day but would present frequently to the neurology clinic due to neck and shoulder pain for 8 years. Prior work up resulted in a normal examination as well as the following normal work up: creatinine kinase, electrodiagnostic testing, magnetic resonance imaging of the brain, lumbar spine, cervical spine, and muscle biopsy of the left deltoid.

In her 50's, her symptoms progressed, and she was working with difficulty. She could not climb stairs or open jars. She could not wash her hair without support, and she was unable to lift heavy objects over her head. She denied any paresthesias or numbness. She noticed some muscle twitching around her eye, triceps, and cheek. She was constipated, and developed intermittent fecal and urinary incontinence. She would also get painful cramps at night.

**Figure 1:**
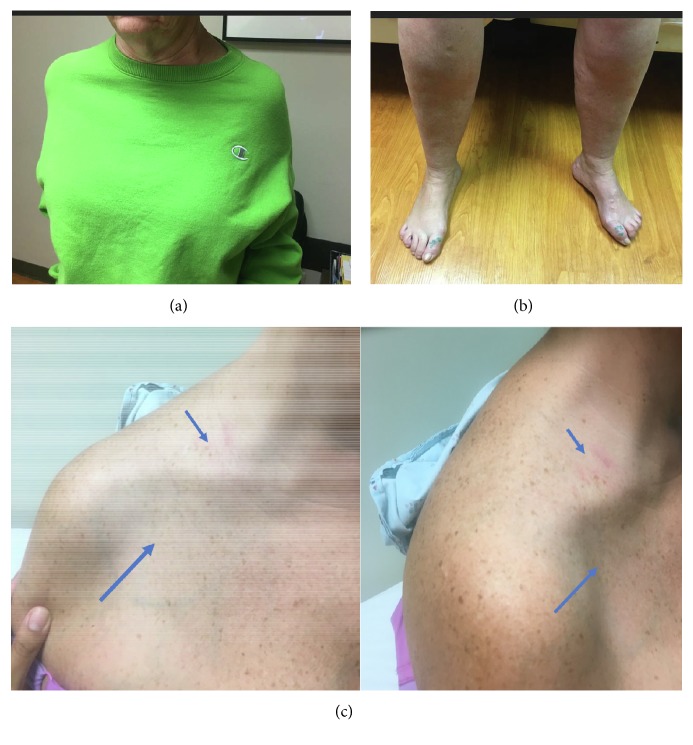
Slanted shoulders seen in the proband (a); asymmetric pes cavus in left foot (b); and trapezius and pectoralis atrophy seen in the proband (arrows) (c).

Upon neurological examination, she presented with slanted shoulders (Figure 1(a)), and asymmetric high arches and hammer toes on the left more than the right (Figure 1(b)). Strength examination (MRC scale) demonstrated trapezius and pectoralis atrophy in the area of her neck pain and weakness (R/L) 0/0 (Figure 1(c)). She was unable to raise her arms greater than 90 degrees over her head. She needed to lean forward to rise from a chair. Vibratory sense, joint position sense, and pinprick were decreased distally in the lower extremities. Deep tendon reflexes were normal and there was no tremor. Cerebellar testing was normal. She had a mildly positive Romberg sign with swaying. Gait was wide-based and she was unable to stand on her toes or heels. Cranial nerve examination and memory testing were normal. There were no fasciculations or upper motor neuron signs on exam.

Repeat laboratory testing included a normal creatine kinase, thyroid stimulating hormone, copper, methylmalonic acid, sedimentation rate, and a normal alkaline phosphatase level. She had a normal sleep study, normal renal ultrasound, and mildly abnormal cardiac stress test with mild ischemia of the anterior cardiac wall. She had a normal pulmonary function test at age 58.

## 3. Electrodiagnostic Testing

Her electrodiagnostic examination changed over time. At age 54, her electrodiagnostic testing was reportedly normal. At age 58, her repeat testing showed an axonal motor neuropathy, neurogenic potentials (large amplitude motor unit potentials with reduced recruitment) in the tibialis anterior bilaterally. Bilateral peroneal motor responses had a reduced amplitude; left peroneal motor response had a prolonged distal motor latency. Nerve conduction velocities were normal except in the right median motor response was mildly slow with a sensory nerve conduction velocity of 42 meters per second at the wrist suggesting a possible superimposed carpal tunnel syndrome. The ulnar, radial, and bilateral sural sensory responses were normal.

## 4. Muscle Biopsy

**Figure 2:**
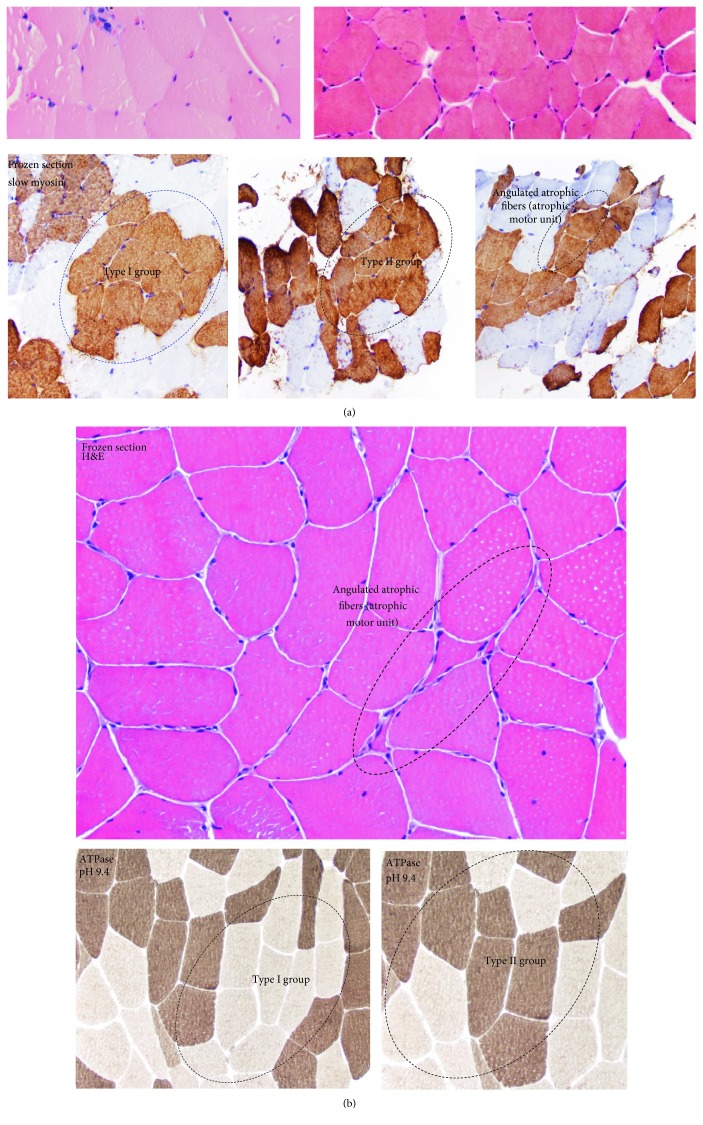
Muscle biopsy of the left deltoid.

Muscle biopsy of the left deltoid done at age 54 was reportedly normal upon repeated discussions; after genetic testing results were positive, the muscle biopsy reading changed slightly with the recognition of a rare atrophic motor unit and a regenerating fiber (Figure 2(a)). The muscle biopsy showed normal fiber type distribution using immunoperoxidase stains for slow and fast myosin heavy chains. There were no target fibers, inflammation, necrosis, regeneration, endomysial fibrosis, inclusion bodies, rimmed vacuoles, or ragged-red fibers. The older brother's muscle biopsy showed angulated atrophic fibers, type 1 and type II group atrophy (Figure 2(b)).

## 5. Family History

**Figure 3:**
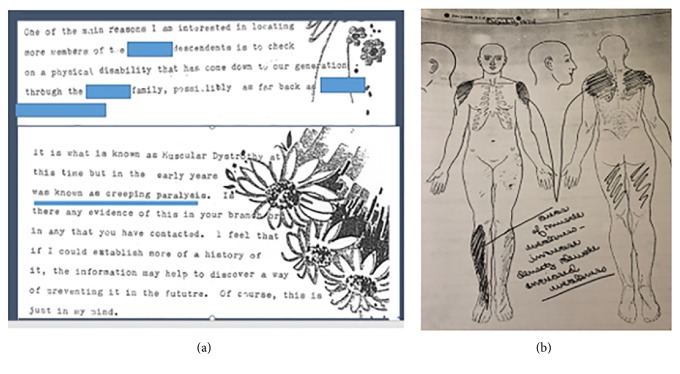
1978 letter from grandmother describing “creeping paralysis” (a) and grandmother's exam shaded in at University of Iowa in 1974 depicting a “scapuloperoneal syndrome” (b).

**Figure 4:**
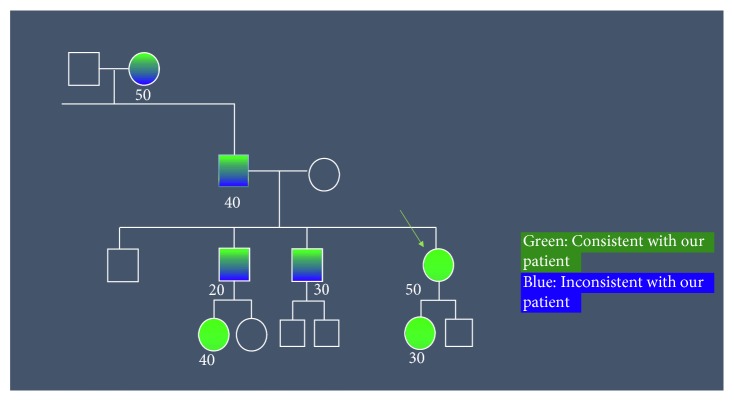
Family pedigree with features consistent and inconsistent with our patient.

Further investigation of the family history revealed a strong desire for an answer over multiple generations (Figure 3(a)). There was also a very complicated pedigree with variable features (Figure 4). Her paternal grandmother had scapuloperoneal weakness with an onset at age 50 (Figure 3(b)). A summary of the family history revealed variable features in almost every family member except for the autosomal dominant scapuloperoneal weakness (Table 1).

## 6. Genetic Testing

For many generations, extensive investigations were performed at multiple institutions revealing that *SMA*, *FSHD*, *SOD1*, and *C9orf72* testing was negative. Limb girdle muscular dystrophy testing revealed a *DYSF* (c.3268C>T, p.R1090C) variant for an autosomal recessive condition. Research exome sequencing of the proband's older brother revealed multiple variants of uncertain significance and was still being analyzed when commercial genetic testing panel in the proband and results revealed a pathogenic *VCP* mutation c. 464g>a, p. Arg155His (R^155^H) [7–12]. This segregated appropriately in all affected family members.

## 7. Discussion

The proband and the family's case presented above is not straightforward clinically and adds to the diagnostic challenge that a *VCP* mutation can produce.

The most important diagnostic challenge in this case was that diagnostic investigations were normal in the proband initially; the electrodiagnostic testing changed over time (described previously [1]) and the pathological evaluation changed after the genetic testing came back positive. Exome sequencing was unrevealing and additional family members could not be obtained easily as presentations in family members were variable and inconsistent. The proband struggled for many years due to the difficulty in obtaining a diagnosis; this difficulty has been described to occur frequently in *VCP* mutations [13].

Another diagnostic challenge was obtaining the family pedigree in a family that had difficult-to-localize neurological evaluations. Despite the scapuloperoneal syndrome seen in every affected family member, the other investigations including electrodiagnostic and muscle biopsy investigations were variable ranging from mixed neuropathic and myopathic features. This has been reported previously with a study on the R^155^H *VCP* mutation showing both myopathic and neurogenic features on EMG [14]. The study also suggested that 60% of muscle biopsies showed nonspecific myopathic changes and did not have to show inclusion body myopathy [14]. There was a large variability in the creatinine kinase level as well as a large interfamilial and intrafamilial variation in patients with valosin containing protein mutations similar to our family [14]. It seemed that the women had milder later onset symptoms compared to the men; this reasons for this are uncertain.

The resolution of this family's case reflects a new generation of diagnosing neuromuscular diseases; in the setting of a positive family history, neurologists should have a lower threshold to obtain genetic testing in the appropriate clinical context.

**Figure 5:**
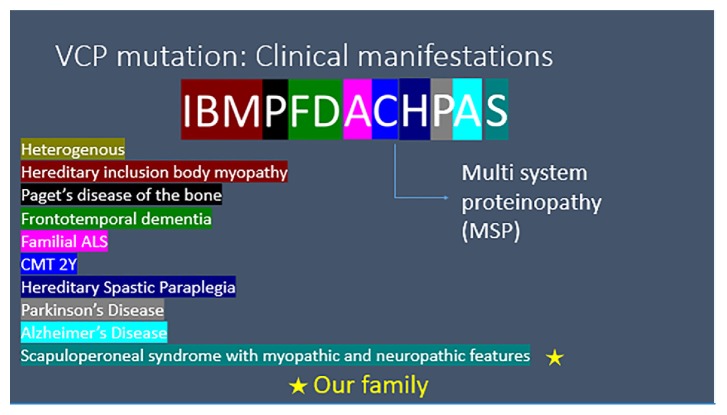
The expansion of the VCP spectrum.

As *VCP* mutations are presenting with more and more varied neurological presentations, the clinical spectrum continues to expand (Figure 5). The scapuloperoneal syndrome itself adds to the clinical spectrum of *VCP* disease (Figure 5). A similar clinical presentation of a scapuloperoneal syndrome with mixed neuropathic and myopathic features was illustrated in a previous case report [1].

Although the homozygous R^155^H/R^155^H mouse model exhibits weakness, myopathic changes on electromyography, accelerated *VCP* disease pathology, Paget disease of the bone, and survival time of less than 21 days, the human manifestations of an individual heterozygous for the R^155^H pathologic gene mutation can present in numerous ways (Figure 5). The reason for this variability could be due to the multiple functions of the *VCP* gene, damaged mitochondria [10, 15], and possibly due to variable penetration.

This case reflects the new term of multi-system proteinopathies [4]. *VCP* mutations can be considered the jack of all trades of neuromuscular medicine—a highly variable mutation that can present with both myopathic and neuropathic weakness including neck pain. When asked to localize in neurology, one may have a hard time figuring out the localization of a *VCP* mutation given its effect on multiple neurological processes and its change in diagnostic evaluations over time.

## Figures and Tables

**Table 1:** Summary of the family history.

Family member	Scapuloperoneal syndrome, age of onset of significant symptoms	Pes Cavus	Reflexes	Dyspnea	Incontinence	CK elevated	EMG abnormal	Muscle biopsy	Dementia
Grandmother	50	Present	Brisk, ankle reflex absent				Neurogenic	Neuropathic and Myopathic	Present at old age per family but not formally tested
Father	40		Absent				Neurogenic	Neuropathic	
Proband (confirmed by genetic testing)	50	Present	Normal		Present		Neurogenic	“Normal” initially but reread to suggest neuropathic	
Proband's brother #1	20		Absent	Present		Present		Neuropathic	
Proband's brother #2	30		Absent				Neurogenic	Myopathic	
Proband's daughter (confirmed by genetic testing)	Shoulder and neck pain, age 30								
Proband's niece (confirmed by genetic testing)	Shoulder and neck pain, age 40								
